# Safe and Effective Administration of Caplacizumab in COVID-19-Associated Thrombotic Thrombocytopenic Purpura

**DOI:** 10.3390/hematolrep15030046

**Published:** 2023-07-20

**Authors:** Antonella Bruzzese, Ernesto Vigna, Dario Terzi, Sonia Greco, Enrica Antonia Martino, Valeria Vangeli, Francesco Mendicino, Eugenio Lucia, Virginia Olivito, Caterina Labanca, Rosellina Morelli, Antonino Neri, Fortunato Morabito, Francesco Zinno, Antonio Mastroianni, Massimo Gentile

**Affiliations:** 1 Hematology Unit, Azienda Ospedaliera Annunziata, 87100 Cosenza, Italy; 2 Immunohaematology Section, Annunziata Hospital, 87100 Cosenza, Italy; 3 Infectious & Tropical Diseases Unit, Annunziata Hospital, 87100 Cosenza, Italy; 4 Medicine Department, “Annunziata” Hospital of Cosenza, 87100 Cosenza, Italy; 5 Scientific Directorate IRCCS of Reggio Emilia, 42123 Reggio Emilia, Italy; 6 Biotechnology Research Unit, AO of Cosenza, 87100 Cosenza, Italy; 7 Department of Pharmacy, Health and Nutritional Science, University of Calabria, 87036 Rende, Italy

**Keywords:** COVID-19, TTP, caplacizumab

## Abstract

Thrombotic thrombocytopenic purpura (TTP) is a potentially life-threatening, rare acute thrombotic microangiopathy (TMA), caused by a severe ADAMTS13 deficiency. As the COVID-19 pandemic rapidly spread around the globe, much data about the pathogenicity of this virus were published. Soon after the detection of the first cases of COVID-19, it was clear that there was a wide range of COVID coagulopathy manifestations, such as deep venous thrombosis, pulmonary thromboembolism, and thrombotic microangiopathies. In the literature, little data have been reported about the association between TTP and COVID-19, and the treatment of COVID-19-associated TTP is still under debate. Here we present the case of a 46-year-old woman who developed a COVID-associated TTP, successfully treated with plasma exchange (PEX), steroids, and caplacizumab.

## 1. Introduction

Thrombotic microangiopathy (TMA) describes a pathological and clinical entity characterized by the formation of thrombi in large and small vessels and microangiopathic hemolytic anemia (MAHA) with thrombocytopenia. Different entities present with TMA findings; sometimes the differential diagnosis is challenging [1].

Thrombotic thrombocytopenic purpura (TTP) is a rare, potentially life-threatening acute thrombotic microangiopathy (TMA) due to a severe deficiency of ADAMTS13 (a disintegrin and metalloprotease with thrombospondin type 1 repeats, member 13), the specific von Willebrand factor (VWF)-cleaving protease [1]. TTP is characterized by MAHA with severe thrombocytopenia, red cell fragmentation, and variable organ ischemia, above all cardiac, renal or neurological [1]. The diagnosis is confirmed by a severe deficiency (<10%) of ADAMTS13 activity [2]. 

In physiologic conditions, under shear forces, VWF unravels and exposes its A1 domain, allowing for interaction with platelets through the GpIb/IX/V complex [3,4,5]. In this conformation, the A2 domain of VWF is elongated and the ADAMTS13 binding sites are exposed for cleavage of VWF multimers [6,7].

When ADAMTS13 is <10%, unusually large von Willebrand factor (ULVWF) multimers can accumulate, with consequent abnormal platelet adhesion and aggregation, leading to disseminated microthrombi and organ ischemia overall in the brain, heart, and kidneys [1,8].

Congenital TTP, also known as Upshaw–Schulman syndrome, is characterized by a persistent severe ADAMTS13 deficiency (<10%), due to biallelic pathogenic mutations in the *ADAMTS13* gene [3]. On the other hand, acquired TTP is caused by ADAMTS13 deficiency mediated by autoantibodies and can be further subdivided into primary iTTP, when there is no obvious associated disorder, and secondary iTTP, when an associated condition can be identified [8].

The majority of iTTP cases are primary. The most common causes of secondary iTTP are infections (though the best evidence is its association with HIV [9]) acute stressors, many drugs, and autoimmune conditions [8].

It has to be noted that both primary or secondary iTTP require prompt therapy. Secondary iTTP typically also requires treatment of the underlying condition in addition to standard TTP therapies.

As the COVID-19 pandemic rapidly spread around the globe, much data about the pathogenicity of this virus were published. Soon after the detection of the first cases of COVID-19, it was clear that this virus leads to a wide range of coagulation alterations, such as deep venous thrombosis, pulmonary thromboembolism, and thrombotic microangiopathies. In these cases, hypercoagulability is an essential hallmark of inflammation, and proinflammatory cytokines lead to platelet and coagulation cascade activation [10,11].

Few data have been reported about the association between TTP and COVID-19. The presentation of COVID-19-associated TTP seems atypical, with no patients reporting fever and only 27.3% having neurological symptoms. Moreover, no relation between the severity of infection and the development of TTP has been noted, and the mean time from COVID-19 symptoms to TTP diagnosis is around ten days [12].

## 2. Case Report

Here we report the case of a 46-year-old woman who presented to the emergency department because of thrombocytopenia at routine exams, without any symptoms. During hospitalization, in a few hours, she developed fever, delirium, agitation, and aphasia. The blood tests showed a worsening of anemia and thrombocytopenia: hemoglobin 8.5 g/dL, platelet count 8000/mmc, white blood cells 7800/mmc, neutrophils 5000/mmc, lymphocytes 1800/mmc, creatinine 0.83 mg/dL, LDH 1037 U/L, total bilirubin 2.02 mg/dL, prothrombin time 12.4 s, international normalized ratio (INR) 1.09. The direct and indirect antiglobulin tests were both negative. The morphologic examination of the venous blood smear revealed the presence of numerous schistocytes (>5%). Since the PLASMIC score value was 7, according to the literature, the patient was judged likely to have an acquired TTP.

Considering the severity of clinical presentation and the high PLASMIC score level, we decide, even in the absence of ADAMTS13 activity data, to start immediate treatment for TTP with daily plasma-exchange (PEX) associated with methylprednisolone 1 mg/kg and Caplaicizumab. Nevertheless, the ADAMTS13 activity tested in our center by ELISA (enzyme linked immunosorbent assay) assay, resulted undetectable, confirming the diagnosis of TTP.

After the first PEX, the persistence of mild fever and consideration of the pandemic of SarsCoV2, prompted us to perform a COVID-19 test, which resulted positive. Thus, the patient was admitted to the infectious disease department. During the hospitalization, PEX, steroids, and caplaicizumab were all continued, regularly.

Considering the symptomatic COVID infection, treatment with a high dose of steroids was initiated. Moreover, infectious disease specialists and hematologists decided collectively to start antiviral therapy with remdesevir 100 mg/day for 4 days, simultaneously with treatment for TTP. Four days after the beginning of treatment, the platelets level rose up >50,000/mmc and cardioaspirin was added to the treatment program.

After 7 days, we observed a complete clinical and biological remission of the disease, so the patient started tapering from PEX.

After 17 days after the first COVID-19 detection, a PCR test showed complete virus clearance, and the patient was discharged with TTP remission.

## 3. Discussion

SARS-CoV-2 has also been associated with TTP. Literature review shows that there have been some differences in the presentation, diagnostics, and management of such patients [12,13,14,15,16,17]. In most of the previously reported cases, patients had other comorbidities such as diabetes, hypertension, or a history of previous malignancy. In all of the cases, patients were younger than 60 years old, and most were female. Concerning the symptoms, in most previously reported cases, patients present with thrombocytopenia and haemolytic anemia, while a severe clinical presentation with renal or neurological disturbance is rare. Conversely, in our case, the patient presents with severe neurologic impairment with fever, delirium, agitation, and aphasia.

Recently, a similar case was described. Mushtaq and colleagues reported the case of a 49-year-old man who presented with fever and severe neurologic impairment with a Glasgow Coma Scale (GCS) score of three. However, in this latter case, the laboratory parameters was not so altered as in our case (mild anemia and thrombocypoenia and normal value of total bilirubin) [18].

Concerning the diagnosis, most of the cases reported use ADAMTS13 activity to establish the diagnosis of TTP. However, in few cases, when ADAMTS13 activity is not immediately available, the PLASMIC score has been used [15,17].

According to the last ISTH guidelines, the diagnostic pathway for PTT involves laboratory tests for ADAMTS13 activity and antiADAMTS13 antibodies. However, in some cases, ADAMTS13 activity is not available or is available with a delay. In these cases, the guidelines suggest to use a clinical risk assessment model, such as the PLASMIC score or the French score [19,20,21].

In our country, the most used model is the PLASMIC score, which gives one point to each variable: (a) platelets <30 × 109/L, (b) haemolysis (defined as reticulocyte count >2.5% or undetectable haptoglobin or indirect bilirubin >2.0 mg/dL), (c) no active cancer, (d) no history of solid-organ or stem-cell transplant, (e) prothrombin time >15 s, (f) creatinine <2.0 mg/dL, (g) mean corpuscular volume of <90 fL. A score of 0–4 is labeled as low risk, associated with no risk of severe ADAMTS13 deficiency, while a score of 5 is labeled as intermediate risk with a 6% chance of severe ADAMTS13 deficiency. A score >5 points is labeled as a high-risk group and is associated with a 72% chance of ADAMTS13 deficiency [20].

In our department, we have the chance to assess ADAMTS13 activity. However, in the present case, the patient arrived during the evening with a catastrophic clinical presentation, with a PLASMIC score of 7. So, we decided to start the PTT treatment without waiting for ADAMTS13 activity.

Concerning treatment, we have to underline that all patients receive two different therapeutic strategies: treatment for COVID-19 and treatment for TTP, both equally important and urgent.

Concerning the treatment for COVID-19, not all case reports report the treatment strategy used. From the literature data available, it emerges that during a PPT episode, different treatments have been reported in the literature, also reflecting the treatment most suggested by the scientific community at that particular moment, with inferteron and dexamethasone used in the first cases described and remdesevir in the latter ones.

In our case, we use the most widely applied strategy in Europe with remdesevir 100 mg/kg day for 4 days, simultaneously with treatment for TTP. Concerning the vaccination, our patient received three doses of vaccination, the last one six months before the infection.

Concerning the treatment for TTP, literature shows that most cases have been treated with PEX and glucocorticoids, apart from one case where due to lack of PEX, IVIG and fresh frozen plasma were used [14]. 

The use of monoclonal antibodies has become common in treating COVID-19-associated TTP. Rituximab and caplacizumab have been used successfully in these patients [12,13,14,15,16,17,18].

Literature data about the best management of TTP during COVID infection are scarce. Recently, a case series showed that patients with TTP and COVID infection respond to traditional treatment of TTP, such as plasma exchange (PEX) with fresh frozen plasma (FFP), FFP infusion, and Rituximab (RTX); conversely, fewer data are available about the use of caplaicizumab [14].

In the literature, different cases of COVID-19-associated microangiopathies have been reported, with different management. In some cases, PEX has been associated with intravenous immunoglobulins (IVIG); this combination has been reported as a safe and effective strategy [22,23].

Some reports suggested that anticoagulation with unfractionated heparin or low-molecular-weight heparin reduced the 28-day mortality rate [24]. In a Chinese report, administering low-molecular-weight heparin in COVID patients reduced IL-6 levels and improved anti-coagulation indices [25].

Caplaicizumab is a monoclonal antibody directed against VWF. In the phase 3 HERCULES trial that enrolled patients with both a first episode or recurrence of TTP, treatment with caplacizumab was associated with a lower incidence of a composite of TTP-related death, recurrence of TTP, or a thromboembolic event during the treatment period compared to a placebo. Moreover, in this study, the median time to normalization of the platelet count was shorter with caplacizumab than with the placebo (2.69 vs. 2.88 days), and patients who received caplacizumab were 1.55 times as likely to achieve a normalization of platelet count as those who received a placebo within 5 days. The percentage of patients with a composite outcome event (recurrence or thromboembolic event or death) was 74%, 12%, and 49% in the caplacizumab and placebo group, respectively. Refractory disease developed in no patients in the caplacizumab group and in three patients in the placebo group. Moreover, patients in the caplacizumab group experience fewer days of hospitalization and needed less plasma exchange than those who received placebo. The most common adverse event was mucocutaneous bleeding, which was reported in 65% and 48% of patients in the caplacizumab and placebo group, respectively. During the trial treatment period, three patients in the placebo group died. One patient in the caplacizumab group died from cerebral ischemia after the end of the treatment period. The data from the HERCULES trial led to the FDA and EMA approval of Caplaicizumab for TTP’s first episode or recurrence [26]. Unfortunately, both the TITAN and HERCULES trials completed enrollment before the COVID 19 pandemic. Therefore, no patients with COVID19 infection and TTP have been enrolled in the trial and data about the use of caplacizumab in COVID19 TTP derives from case reports.

Chaudhary et al. reviewed 11 cases of COVID-19-associated TTP published in the literature in 2022 and found that females were more likely to develop this disease combination. Dyspnea was the most common symptom at presentation, followed by neurological symptoms, and none of the patients experienced a febrile illness before TTP began. The mean duration from the onset of COVID-19 to the diagnosis of TTP was 10 days. In no case did the onset of TTP precede the first detection of COVID-19. In this review, all 11 cases underwent PEX, with an average of 12 sessions for each patient. All patients received steroids. Of the 11 cases, 6 received RTX and 3 received caplaicizumab, in 2 cases associated with RTX. In 2 cases, caplacizumab was added owing to an inadequate response after PEX and RTX. All cases of COVID19-associated TTP treated with caplacizumab had a favorable outcome with no death reported and no early relapse during the follow up [12].

It has been suggested that, in COVID associated TTP, the complement cascade activation plays a crucial role, leading to a complement mediated endothelial injury causing micro-thrombosis. In these cases, treatment with complement inhibitors may be useful [17,27,28].

Many viral infections could give rise to a secondary immune TTP, likely due to both the development of autoantibodies against ADAMTS13 and direct endothelial injury [18,19]. Concerning COVID-19 infection, it has been supposed that the hypercoagulable state observed in infected patients is partially due to endothelial cell injury mediated by entry via ACE-2 receptors [20]. Moreover, it seems that COVID-19 inflammation could lead to a reduction in ADAMTS13 activity and an increase in fibrinogen, D-dimer, vWF, Factor VIII, fibrinogen, and D-dimer levels [21,22]. All these mechanisms may trigger and maintain COVID-19 associated TTP, partially explaining the high percentage of refractoriness to standard treatment. Recently, two studies showed a relation between a reduction in ADAMTS13 activity and the severity of COVID-19 illness [14,21]. On the other hand, most reviewed cases (81.8%) with confirmed TTP had undetectable ADAMTS13 activity (i.e., 10%) without clinically severe COVID-19 infection.

Unfortunately, a correct diagnosis of TTP is not easy in COVID-19 infections; in fact, the combination of severe thrombocytopenia and hemolysis could be representative of other TMA (such as HUS or drug-related TMA) or disseminated intravascular coagulation (DIC), which are also common in COVID-19 infections [23]. Consequently, it is clear that a rapid diagnosis of TTP is necessary to reduce mortality, reported above 90% in untreated patients and 10% in ones who receive a prompt treatment [24].

Moreover, all cases of COVID-19 associated TTP treated with caplacizumab had a favorable outcome.

No data are available about antiviral treatment with remdesevir in patients with COVID-19-related TTP. In the present case, in agreement with infectivologists, we decided to start an antiviral therapy to stop the progression of the COVID-19 disease and remove the trigger of TTP.

## 4. Conclusions

In conclusion, the best management of TTP in COVID-19 remains an unmet clinical need. The present case highlights the most important unmet questions about the treatment of both COVID-19 infection and TTP.

## Data Availability

No new data were created for this manuscript.
